# The Relationship Between Peritumoral Brain Edema and the Expression of Vascular Endothelial Growth Factor in Vestibular Schwannoma

**DOI:** 10.3389/fneur.2021.691378

**Published:** 2021-08-09

**Authors:** Hong-Hai You, Xiao-Yong Chen, Jin-Yuan Chen, Yue Bai, Fu-Xiang Chen

**Affiliations:** ^1^Department of Neurosurgery, The First Affiliated Hospital of Fujian Medical University, Fuzhou, China; ^2^Department of Ophthalmology, The First Affiliated Hospital of Fujian Medical University, Fuzhou, China

**Keywords:** vestibular schwannoma, peritumoral brain edema, vascular endothelial growth factor, microvessel density, edema index

## Abstract

**Objective:** This study aimed to explore the potential mechanism of peritumoral brain edema (PTBE) formation in vestibular schwannoma (VS) by detecting intra-tumoral vascular endothelial growth factor (VEGF) expression.

**Methods:** Between January 2018 and May 2021, 15 patients with PTBE and 25 patients without PTBE were included in the analysis. All patients enrolled in our study underwent surgery in our institution. Expression level of VEGF and microvessel density (MVD) between the two groups were analyzed. Edema index (EI) of each patient with PTBE was calculated.

**Results:** In the PTBE group, the average of EI was 1.53 ± 0.22. VEGF expression levels were significantly enhanced in the PTBE group compared with the non-PTBE group (*p* < 0.001). The expression level of VEGF in the PTBE group and non-PTBE group was 1.14 ± 0.21 and 0.52 ± 0.09, respectively. Similarly, there were significantly different amounts of MVD in the two groups (*p* < 0.001). The amount of MVD in the PTBE group and non-PTBE group was 11.33 ± 1.59 and 6.28 ± 1.77, respectively. Correlation analysis showed a highly significant positive correlation between VEGF and MVD (*r* = 0.883, *p* < 0.001) and VEGF and EI (*r* = 0.876, *p* < 0.001).

**Conclusion:** Our study confirmed the close relationship among VEGF expression, tumor angiogenesis, and formation of PTBE in VS patients. It may be possible to develop new effective therapies to attenuate PTBE in VS for alleviation of symptoms and reduction of postoperative complication.

## Introduction

Vestibular schwannoma (VS) is common benign tumor of the central nervous system accounting for 8.43% of the total (1), which is mainly located in the cerebellopontine angle. Peritumoral brain edema (PTBE) is a common sign of intracranial tumor but is less common in VS. Nevertheless, there are still a proportion of VS patients accompanied with PTBE, about 5–10% (1, 2). Severe PTBE could cause suffering for patients and difficulties for surgeons, including aggravation of clinical symptoms, increase of surgical difficulties, and increase of postoperative complication risk.

Currently, the mechanism of PTBE formation is still unclear. Some scholars suggested that vascular endothelial growth factor (VEGF) played an important role in the process of PTBE formation in many intracranial tumors (3, 4). However, it is unknown whether VEGF plays a role in PTBE formation of VS. In our study, we aimed to explore the potential mechanism of PTBE formation in VS by detecting intra-tumoral VEGF expression.

## Materials and Methods

This study was performed at the First Affiliated Hospital of Fujian Medical University. All procedures were executed with the approval of the ethics committee of the First Affiliated Hospital of Fujian Medical University and the patients' written informed consent. Between January 2018 and May 2021, 180 patients with VS in our institution who were willing to receive surgical therapy were considered for inclusion. Before surgery, all patients underwent plain and enhanced magnetic resonance imaging (MRI) examination. Of this patient cohort, 15 patients with PTBE were included in the PTBE group. As a control, 25 patients without PTBE were randomly selected and assigned to the non-PTBE group. All tumor specimens from the patients in the PTBE group and the non-PTBE group were obtained for further analysis after surgery and confirmed as VS based on pathological assessment.

### Calculation of Edema Index

**Figure 1:**
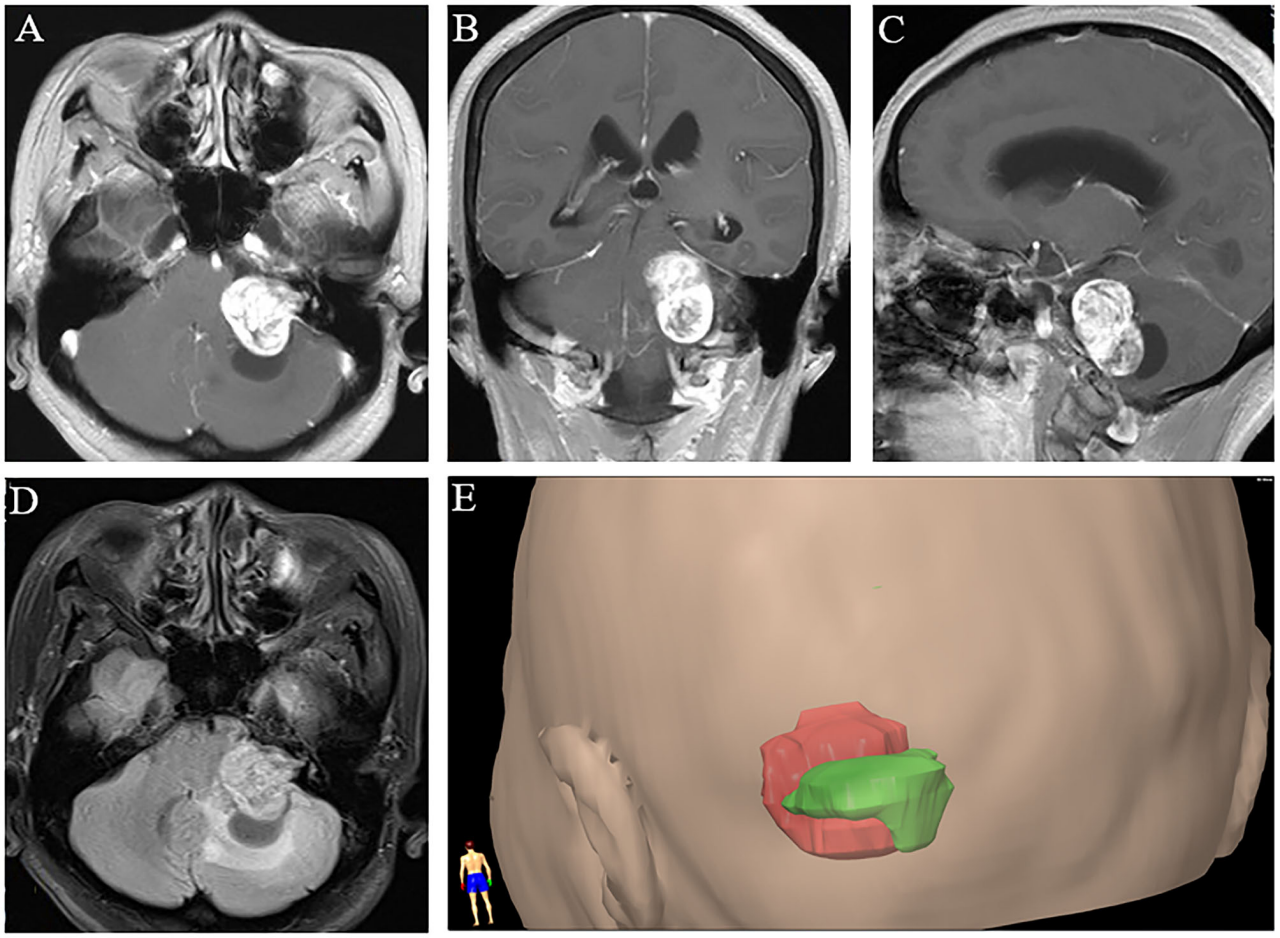
Three-dimensional magnetic resonance imaging reconstruction of vestibular schwannoma with peritumoral brain edema. **(A–C)** Post-contrast axial, coronal, and sagittal T1-weighted images. **(D)** T2-weighted fluid-attenuated inversion-recovery image. **(E)** A representative image of three-dimensional reconstruction with tumor in red and peritumoral edema in green.

The boundary of the VS was determined by contrast-enhanced T1-weighted imaging (Figures 1A–C). Using fluid-attenuated inversion-recovery (FLAIR) images (Figure 1D), we determined the boundary of PTBE. For each case in the PTBE group, three-dimensional reconstruction of the tumor with PTBE was performed to calculate the volume of tumor and edema based on neuronavigation workstation (iCranial v.3.0 stereotacxy; BrainLab, Munich, Germany) at our department (Figure 1E). The same approach was applied to calculate the volume of tumor in the non-PTBE group. The edema index (EI) was used to evaluate the severity of PTBE, which was defined as previously reported (5): (Vedema + Vtumor)/Vtumor. The degree of PTBE was graded as follows: 1, absence of edema; 1–1.5, mild; 1.5–3.0, moderate; and >3.0, severe.

### Identification of Microvessel Density by CD34 Staining

**Figure 2:**
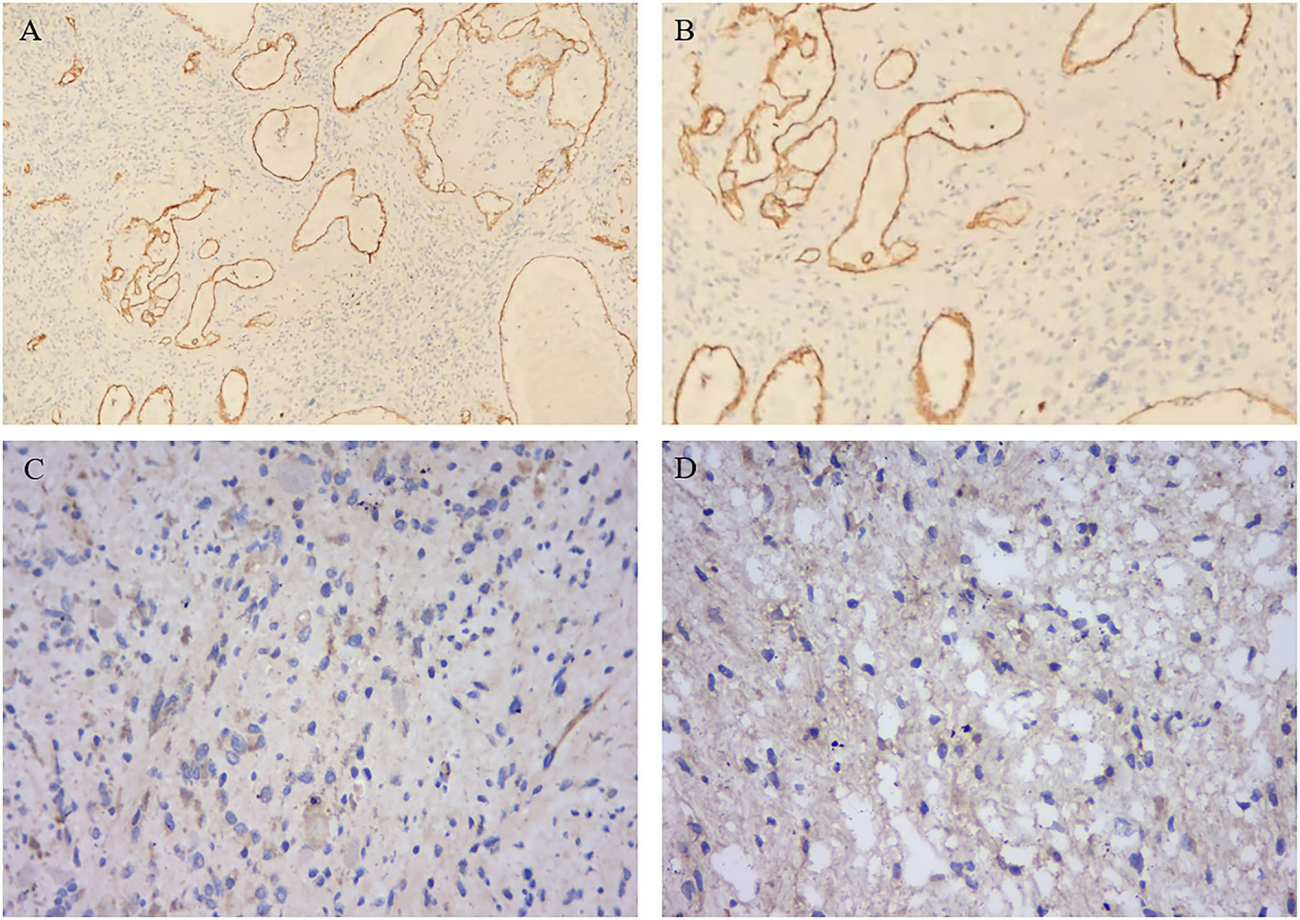
Representative immunohistochemical staining of microvessel density and VEGF in PTBE group. **(A)** Low-magnification view of MVD. **(B)** High-magnification view of MVD. **(C)** Low-magnification view of VEGF staining. **(D)** High-magnification view of VEGF staining. MVD, microvessel density; VEGF, vascular endothelial growth factor; PTBE, peritumoral brain edema.

By Weidner's criteria based on CD34 immunohistochemical staining (6), the microvessel density (MVD) was determined. First, the tissue sections were observed at low magnification (×100) to select the region of the highest neovascularization, which was also defined as “hot spot” (Figure 2A). Then, the number of microvessels of each “hot spot” was counted under a microscope at ×200 magnification (Figure 2B). The mean value of MVD in three randomly selected fields was finally calculated. Any single-stained endothelial cell or cluster of endothelial cells with or without vessel lumen, which was clearly separated from tumor cells, adjacent microvessels, and connective tissue, was recognized as a single, countable microvessel.

### Immunohistochemical Staining of Vascular Endothelial Growth Factor

Surgically resected tumor specimens were fixed in 10% formaldehyde, and serial sections were prepared after paraffin embedding. Then, standard hematoxylin and eosin (H&E) staining and streptavidin–biotin peroxidase (SP) immunohistochemical technique were performed. Diaminobenzidine (DAB) was used as chromogen to perform SP detection for evaluating VEGF expression (Figures 2C,D). Based on the staining intensity, the VEGF expression was scored as previously described (grade 1, negative; grade 2, weak; grade 3, moderate; and grade 4, strong staining) (7), which corresponds to the percentage of stained tumor area at ×200 magnification (1, <5%; 2, 5–20%; 3, 21–50%; and 4, >50%).

### Western Blotting Analysis

Western blotting detection kits, which were purchased from Wuhan ServiceBio Technology Co., Ltd. (Wuhan, China) were used in strict accordance with the manufacturer's recommendations. Briefly, total cell protein was extracted and measured by ultraviolet spectrophotometer. Samples were denatured by boiling for 5 min and then electrophoresed in 12% sodium dodecyl sulfate (SDS)–polyacrylamide gels. Proteins were transferred to NC membranes and blocked with 5% skimmed milk for 1 h at room temperature. The primary antibodies were diluted in TBST with 5% skimmed milk or 5% bovine serum albumin (BSA) and incubated overnight at 4°C. Membranes were washed by TBST for three times (5 min per time). The secondary antibodies (diluted 1:3,000 in TBST) were incubated at room temperature for 30 min. Then, they were re-washed by TBST for three times (5 min per time) and reacted to enhanced chemiluminescence (ECL) solution for 1–2 min. After that, the membranes were exposed, developed, and fixed on X-ray films for further scanning and archiving. The optical density values of the target band were analyzed by AlphaEaseFC software.

### Statistical Analysis

All analyses were performed with SPSS version 22.0 (SPSS, Inc., Chicago, IL, USA). Continuous variables, presented as mean ± standard deviation or median (inter-quartile range), were analyzed by two-sample *t*-test or non-parametric test, respectively. Pearson's correlation analysis was adopted for determining correlation. A *p* < 0.05 was considered as statistically significant.

## Results

### Clinical Parameters and Tumor Characteristics

As previously mentioned, we analyzed 40 cases for this study. The clinical data of all patients were complete and presented in Table 1. Overall, 17 were male and 23 were female with a median age of 53.9 (39.4–62.1) years. There were no statistical differences between the two groups in age (*p* = 0.727), sex (*p* = 0.283), and sample location (*p* = 0.622).

**Table 1:** Clinical data obtained for each of the 40 patients.

**Case no**.	**Age** **(year)**	**Sex**	**Sample**	**Location**	**Peritumoral edema**
1	56.5	M	Vestibular schwannoma	R	Yes
2	50.2	M	Vestibular schwannoma	L	Yes
3	56.6	M	Vestibular schwannoma	L	Yes
4	51.6	F	Vestibular schwannoma	L	Yes
5	75.8	F	Vestibular schwannoma	L	Yes
6	60.0	F	Vestibular schwannoma	L	Yes
7	56.6	F	Vestibular schwannoma	R	Yes
8	25.3	M	Vestibular schwannoma	L	Yes
9	43.2	M	Vestibular schwannoma	L	Yes
10	27.2	F	Vestibular schwannoma	L	Yes
11	53.2	M	Vestibular schwannoma	R	Yes
12	64.6	F	Vestibular schwannoma	R	Yes
13	33.3	M	Vestibular schwannoma	L	Yes
14	65.3	M	Vestibular schwannoma	R	Yes
15	38.4	F	Vestibular schwannoma	R	Yes
16	46.0	F	Vestibular schwannoma	R	No
17	53.9	F	Vestibular schwannoma	L	No
18	53.8	F	Vestibular schwannoma	L	No
19	43.5	M	Vestibular schwannoma	R	No
20	56.8	F	Vestibular schwannoma	R	No
21	71.6	F	Vestibular schwannoma	R	No
22	70.4	F	Vestibular schwannoma	L	No
23	24.8	M	Vestibular schwannoma	R	No
24	27.6	F	Vestibular schwannoma	L	No
25	57.8	F	Vestibular schwannoma	R	No
26	28.9	M	Vestibular schwannoma	L	No
27	61.9	F	Vestibular schwannoma	R	No
28	67.1	F	Vestibular schwannoma	R	No
29	26.4	M	Vestibular schwannoma	L	No
30	64.9	F	Vestibular schwannoma	L	No
31	46.1	F	Vestibular schwannoma	L	No
32	50.8	M	Vestibular schwannoma	L	No
33	42.5	F	Vestibular schwannoma	R	No
34	70.2	F	Vestibular schwannoma	L	No
35	70.8	M	Vestibular schwannoma	L	No
36	23.9	M	Vestibular schwannoma	L	No
37	29.2	F	Vestibular schwannoma	R	No
38	59.6	F	Vestibular schwannoma	R	No
39	62.1	M	Vestibular schwannoma	R	No
40	61.4	M	Vestibular schwannoma	L	No

*M, male; F, female; R, right; L, left*.

Tumor volume in the PTBE group was significantly larger than that in the non-PTBE group (17.23 ± 3.54 vs. 14.72 ± 3.47 cm^3^, *p* = 0.035). In the PTBE group, mild edema was present around the lesions in eight cases, while seven cases presented with moderate edema. The maximum EI was 1.82, with an average value of 1.53 ± 0.22. Tumor volume was not statistically correlated with the severity of PTBE (*p* = 0.619).

### Comparison of Vascular Endothelial Growth Factor Expression and Microvessel Density Among the Two Groups

**Figure 3:**
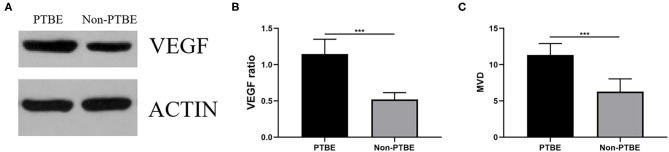
The expression of VEGF and the amount of MVD. **(A)** The bands of VEGF in the two groups by Western blotting analysis. **(B)** Expression analysis showed that patients in the PTBE group had significantly higher expression level of VEGF than those of the non-PTBE group. **(C)** The amount of MVD in the PTBE group was significantly higher than that in the non-PTBE group. ***p < 0.001.

Both groups revealed VEGF protein expression (Figure 3A). VEGF expression levels were significantly enhanced in the PTBE group compared with the non-PTBE group (Figure 3B, *p* < 0.001). The expression level of VEGF in the PTBE group and non-PTBE group was 1.14 ± 0.21 and 0.52 ± 0.09, respectively (Table 2). Similarly, there were significantly different amounts of MVD in the two groups (Figure 3C, *p* < 0.001), with the less amount in the non-PTBE group and the more amount in the PTBE group. The amount of MVD in the PTBE group and non-PTBE group was 11.33 ± 1.59 and 6.28 ± 1.77, respectively (Table 2).

**Table 2:** Comparison of VEGF and MVD in the two groups.

**Parameter**	**PTBE (*n* = 15)**	**Non-PTBE (*n* = 25)**	***p*-value**
VEGF	1.14 ± 0.21	0.52 ± 0.09	<0.001
MVD	11.33 ± 1.59	6.28 ± 1.77	<0.001

*VEGF, vascular endothelial growth factor; MVD, microvessel density; PTBE, peritumoral brain edema*.

### Vascular Endothelial Growth Factor Expression Is Positively Correlated With Microvessel Density and Edema Index

**Figure 4:**
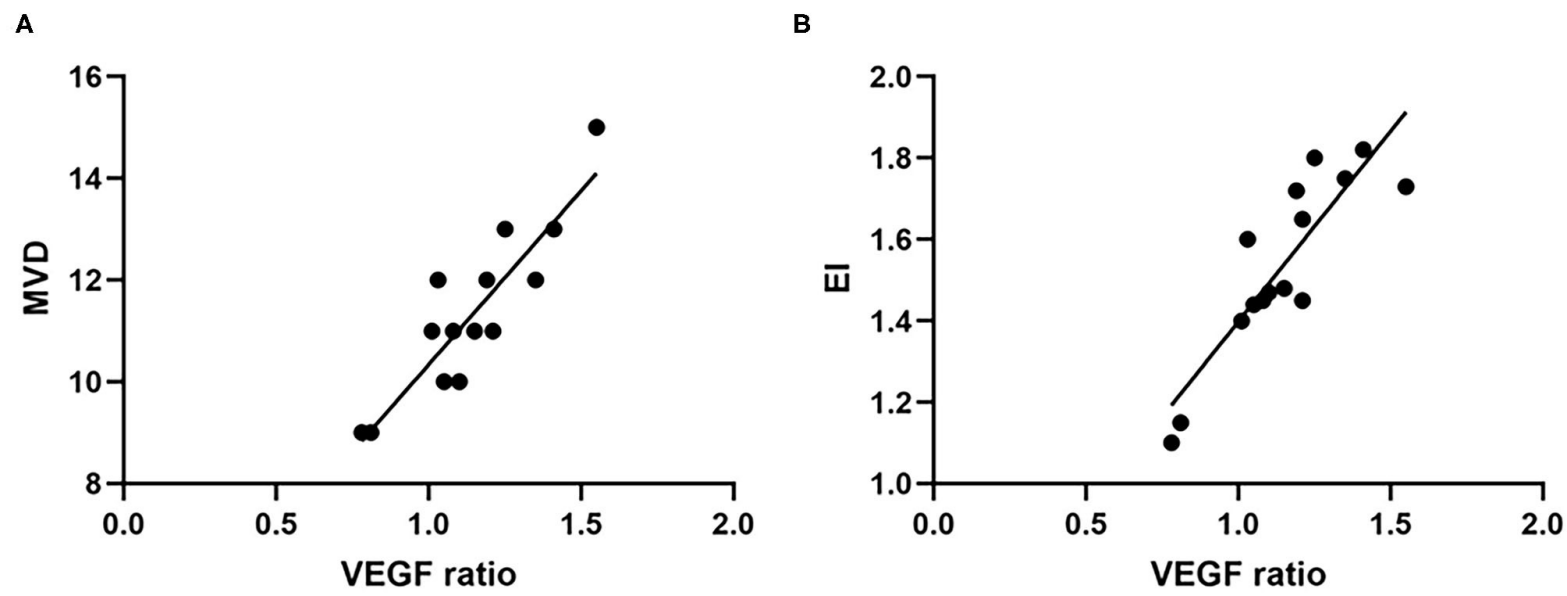
The expression level of VEGF was positively correlated with MVD **(A)** and EI **(B)**. VEGF, vascular endothelial growth factor; MVD, microvessel density; EI, edema index.

In the PTBE group, all samples were positive staining for VEGF. Among them, five patients had a weak staining intensity of VEGF, and the number of patients with a moderate or strong staining intensity was 6 and 4, respectively. There was no significant difference of tumor volume in the patients with different staining intensity (*p* = 0.978). The value of EI was significantly different in the three groups (*p* = 0.001). As displayed in Figure 4, correlation analysis revealed a highly significant positive correlation between VEGF and MVD (*r* = 0.883, *p* < 0.001); similarly, a highly positive correlation between VEGF and EI was confirmed (*r* = 0.876, *p* < 0.001).

## Discussion

The VS can directly compress the cerebellum, the brain stem, and peripheral nerves due to its location in the cerebellopontine angle. The VS on imaging can vary in size and present as a solid, cystic, or solid–cystic lesion. The incidence of VS combined with PTBE is low but not rare. Samii et al. reported 30 cases with PTBE in 605 patients with VS, showing that the incidence of PTBE in VS was only 5% (1), while another study reported a higher incidence of PTBE up to 37% (8). In recent years, with the remarkable development of surgical equipment and improvement of surgical technique, the safety and effectiveness of treatment for VS have substantially improved. Nevertheless, the surgical treatment of patients with VS remains a challenge especially in those with PTBE. The deleterious effects of PTBE are usually presented as a substantial increase in space-occupying effect of VS, including elevation of intracranial pressure and distortion of local structures, subsequently leading to a decrease of cerebral blood flow and a slowed metabolism of nearby cells. In addition, the occurrence of PTBE may contribute to the adherence of VS to the surrounding structures, resulting to the increase of surgical difficulty and postoperative complication rate (2). For example, a previous study reported that VS with PTBE had a worse short-term functional outcome and higher risk of bleeding after surgery (1). Therefore, PTBE in VS is correlated with the clinical course and prognosis of the disease.

Though the basic studies of PTBE in intracranial tumor has achieved promising results, the detailed mechanism of formation and action have not been elucidated so far. Research has suggested that PTBE belonged to vasogenic brain edema (9). Tumor growth and proliferation rely on the formation of its vascular network, which supplies oxygen and nutrients and removes metabolic waste. A high degree of vascularization could promote tumor growth and cause severe PTBE, which has been confirmed in astrocytomas (5). Thus, angiogenesis and anti-angiogenesis factors could regulate neovascularization of tumors and affect PTBE. VEGF is known to be an important angiogenic factor, which promotes the neovascularization and increases vascular permeability resulting in the promotion of tumor growth (10). In the early stages of neovascularization, VEGF modulates endothelial cell proliferation vascular dilation and vascular leakage. And in the later stages, VEGF regulates vessel maturation and stabilization. VEGF expression has confirmed its importance in the formation of PTBE in intracranial tumor, which is gradually becoming a research hot spot (10–13). Nassehi et al. revealed that the VEGF-A pathway may be essential for the formation of PTBE in meningiomas (13). However, there are few previous studies focusing on the association between VEGF and PTBE in VS.

The results of this study showed that tumor volume in the PTBE group was significantly larger than that in the non-PTBE group (*p* = 0.035) but not statistically correlated with the severity of PTBE (*p* = 0.619) and the staining intensity of VEGF (*p* = 0.978), while the value of EI was significantly different in the different grades of staining intensity of VEGF (*p* = 0.001). In addition, the value of MVD and expression of VEGF in PTBE group were significantly higher than those in non-PTBE group. Besides, VEGF expression were positively correlated with MVD and EI. MVD, as a standard measurement of angiogenesis, was widely considered as a prognostic indicator of intracranial tumors, and higher MVD was associated with worse outcome (14–16). The VS combined with PTBE often shows a rich blood supply in imaging examinations and higher volume of intraoperative blood loss. Though some normal tissues may produce VEGF, tumor cells present overexpression of VEGF *via* autocrine or paracrine effects. The increase of neovascularization secondary to overexpression of VEGF could promote the invasive potency of tumor toward the surrounding tissues. On the one hand, the results indicated that VS with PTBE may grow faster than that without PTBE, leading to a higher surgical difficulty; on the other hand, it is suggested that the differential expression of VEGF mainly affects severity of PTBE *via* its effects on vessels. Therefore, we believed that VEGF was an important factor of neovascularization in VS patients and was closely associated with PTBE formation. Without timely surgical resection, PTBE can be worse since more VEGF will be produced by the VS.

**Figure 5:**
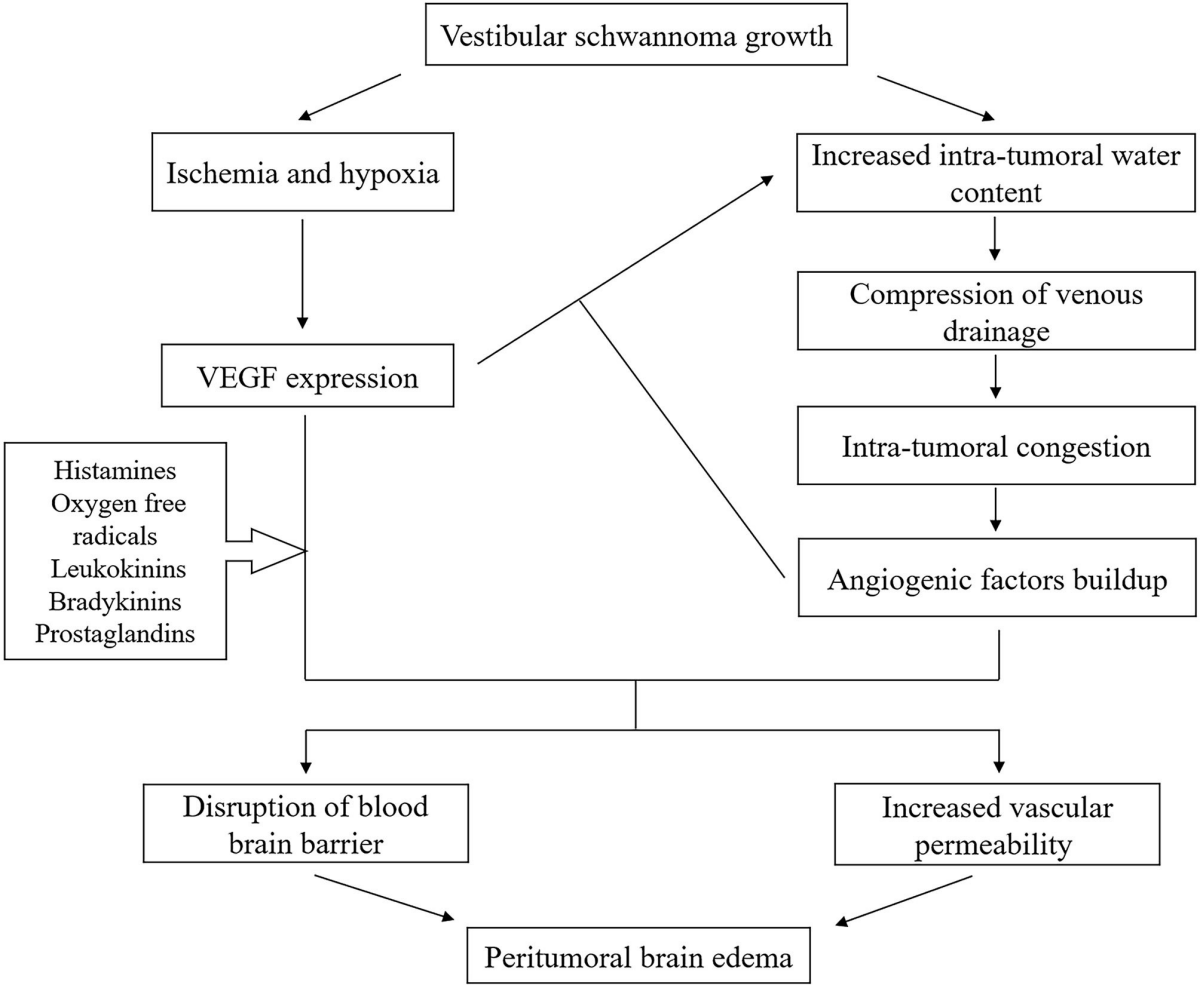
Proposed mechanisms of PTBE formation in VS. PTBE, peritumoral brain edema; VS, vestibular schwannoma.

The VEGF, an endothelial-cell-specific mitogen, was first reported in guinea pig hepatocarcinoma in 1983 (17) and first isolated from pituitary follicular cells in 1989 (18). As a polyfunctional molecule, VEGF is 1,000 times more potent to induce vascular permeability, tumor neovascularization, and PTBE than histamine (19). VEGF is produced and secreted by tumors, acting specifically on endothelial cells to promote vascular endothelial cell proliferation and induce angiogenesis, on the one hand, and increasing vessel permeability to allow the extravasation and deposition of fibrinogen on the other (20). Specifically, VEGF could downregulate the expression of tight junction proteins, leading to increased formation of fenestra and cleft between vascular endothelial cells. Also, it could enhance the vesiculo-vacuolar formation within endothelial cells (21). These alterations may result in the increase of vascular permeability and may ultimately lead to PTBE. The correlation among PTBE, VEGF expression, and tumor neovascularization has already been extensively confirmed (22) and may also exist in VS, which was supported by our study. PTBE is probably a complex multifactorial process and VEGF is supposed to play a crucial role during the process. Since VEGF expression is potentiated by hypoxia and ischemia and PTBE can in turn lead to secondary ischemia, these factors are closely interrelated to each other. Additionally, many cofactors and biochemical vasoactive agents, including histamines, oxygen free radicals, leukokinins, bradykinins, and prostaglandins, may act in synergy with VEGF to promote PTBE formation. As part of the complex network, VEGF interacts with many pathways, including hypoxia-inducible factor, Notch, and aquaporin 4, to modulate angiogenesis and PTBE (23), all of which may be involved in the mechanism of PTBE formation (Figure 5).

Therefore, new treatment approaches of VS with severe PTBE may lie in the reduction of VEGF secretion or the blockage of its receptors (e.g., flk1 and KDR), as they may reduce tumor neovascularization, tumor growth capacity, and tumor invasion capacity. Anti-VEGF monoclonal antibodies may be the most promising therapy, as it could directly target VEGF-expressing tumor cells and may result in tumor cell elimination and PTBE alleviation.

There were also some limitations in our study. First, the number of examined patients was too small due to low incidence of VS with PTBE. Further study with a larger sample size is warranted to overcome this drawback. Second, we did not detect the expression of other cofactors and biochemical vasoactive agents in tumor, which may act in synergy with VEGF to promote PTBE formation. Future work will be dedicated to solve these problems for further exploring the mechanism of PTBE formation in VS.

## Conclusion

Our study confirmed the close relationship among VEGF expression, tumor angiogenesis, and formation of PTBE in VS patients. VEGF could promote angiogenesis of VS, and both of them contributed to the formation of PTBE in VS. Therefore, based on these findings, it may be possible to develop new effective therapies to attenuate PTBE in VS for alleviation of symptoms and reduction of postoperative complication.

## Data Availability Statement

The raw data supporting the conclusions of this article will be made available by the authors, without undue reservation.

## Ethics Statement

The studies involving human participants were reviewed and approved by the Ethics Committee of the First Affiliated Hospital of Fujian Medical University. Written informed consent to participate in this study was provided by the participants' legal guardian/next of kin.

## Author Contributions

H-HY, X-YC, and J-YC were major contributors in concept, design, definition of intellectual content, literature search, experimental studies, data acquisition, data analysis, statistical analysis, manuscript preparation, manuscript editing, and manuscript review of the manuscript. YB and F-XC analyzed and interpreted the data and take responsibility for the integrity of the work as a whole from inception to published article. This work was done in the Department of Neurosurgery, The First Affiliated Hospital of Fujian Medical University, Fuzhou, China. All authors contributed to the article and approved the submitted version.

## Conflict of Interest

The authors declare that the research was conducted in the absence of any commercial or financial relationships that could be construed as a potential conflict of interest.

## Publisher's Note

All claims expressed in this article are solely those of the authors and do not necessarily represent those of their affiliated organizations, or those of the publisher, the editors and the reviewers. Any product that may be evaluated in this article, or claim that may be made by its manufacturer, is not guaranteed or endorsed by the publisher.
